# Temperature-Dependent Excitonic Photoluminescence and Nonlinear Absorption of CdTe Nanocrystal/Polyvinyl Alcohol Films

**DOI:** 10.3390/nano11071761

**Published:** 2021-07-06

**Authors:** Qing Chang, Jingrong Sui, Zhijun Chai, Wenzhi Wu

**Affiliations:** 1 College of Media Engineering, Communication University of Zhejiang, Hangzhou 310018, China; 15846017711@139.com; 2 School of Electronic Engineering, Heilongjiang University, Harbin 150080, China; suijingrong@bj.chinamobile.com (J.S.); 2002076@hlju.edu.cn (Z.C.)

**Keywords:** CdTe nanocrystals, photoluminescence, temperature-dependent, band-edge exciton, nonlinear absorption

## Abstract

The temperature dependence of the excitonic photoluminescence (PL) and nonlinear absorption characteristics of CdTe nanocrystals (NCs)/polyvinyl alcohol (PVA) film are investigated using steady-state/time-resolved PL spectroscopy and Z-scan methods. The excitonic PL peaks of CdTe NCs can be observed at the wavelengths from 560 to 670 nm, with size changes from 2.1 to 3.9 nm. From the temperature-dependent PL spectra, the smaller photon energy of the PL emission peak, the rapidly decreasing PL intensity, and the wider linewidth are observed with increasing temperature from 80 to 300 K. It is revealed that the excitonic PL is composed of both trapped state and band-edge excitonic state, which presents biexponential fitting dynamics. The short-lived species is due to the surface-trapped state recombination in NCs, which has a photogenerated trapped channel and a time-resolved peak shift. The species with a long-lived lifetime is ascribed to the intrinsic excitonic recombination. Through the femtosecond Z-scan method, the nonlinear absorption coefficient becomes smaller with the increase in the size of the NCs. The optical properties of the CdTe NC/PVA film show the potential of II-VI traditional NCs as display and luminescent materials that can utilize the combination of excitonic PL and nonlinear absorption for expanded functionality.

## 1. Introduction

Polymer nanofilms are versatile, functional materials in which nanoscale (1–100 nm) inorganic nanoparticles are dispersed in an organic polymer matrix to display enhanced optical, magnetic, and mechanical characteristics [1,2]. The incorporation of semiconductor nanocrystals (NCs) into a polymer matrix is significantly important in a wide variety of fields for practical, functional applications, such as fiber optics [3], temperature sensors [4], and photovoltaics [5,6], which inevitably change their optoelectronic characteristics with the interaction of host materials under environmental conditions [7]. Bulk CdTe is a semiconductor of the II-VI group with a direct bandgap of 1.40 eV, which possesses an exciton Bohr radius of 6 nm [8]. When the size is smaller or comparable with the exciton Bohr radius, CdTe NCs possess excellent optoelectronic characteristics due to the quantum confinement effect. From a technological perspective, many methods have been applied to synthesize CdTe NCs in thin film form, which includes vacuum evaporation, inkjet printing [9], chemical bath deposition [10], spin coating, electrodeposition, and ion-track templates [11]. However, among these methods, the spin coating technique is suitable for the preparation of polymer-capped II-VI group nanofilm, as it is cost-effective and easy to handle for large-area deposition [12]. The polymer matrix shows no PL, due to its lack of a photoluminescent center. Therefore, it is appropriate to obtain luminescent materials from these kinds of transparent polymers. Moreover, using the coating method, luminescent polymer dots can be similarly excited and could be used for sensor fabrication [13,14]. Important aspects regarding the tremendous optoelectronic applications of NCs are the preservation of the relatively high PL efficiency in the NC polymer films and the uniform distribution of NCs within the films together. To satisfy these demands, a suitable polymer that does not quench the PL intensity of the NCs must be chosen as a matrix material, which also provides a homogeneous NC distribution [9]. Polyvinyl alcohol (PVA) is an important matrix material used for the enhanced emission of aqueous NC LEDs because it can avoid Forster resonance energy transfer (FRET) with enough distance between NCs [3]. To gain a deeper insight of the excitonic recombination and nonlinear absorption into the photophysical process of CdTe NC/PVA films, it is worth discussing at low temperature, where the additional complexity induced by temperature effects is constrained. The introduction of inorganic NCs in organic polymer matrices has attracted considerable attention because the combined advantages of inorganic NCs and organic polymers provides a simple channel to synthesize the processable and stable compound functional materials [15]. Investigations into the optical characteristics of the compound films containing II–VI group NCs have attracted considerable interest due to the potential applications to photoelectric devices [16], but it is rarely reported for the luminescent and nonlinear absorption of CdTe NC/PVA films.

In our investigation, we discuss the temperature-dependent, steady-state, and time-resolved PL properties of CdTe NC/PVA films compared with pure CdTe NCs. The temperature dependence on steady-state PL peak energy, intensity, and linewidth of the NCs are investigated. The exciton binding energy, optical phonon-exciton coefficient, and longitudinal optical-phonon energy are calculated by fitting to the temperature-dependent excitonic PL spectra for CdTe NC/PVA films. As size decreases, the nonlinear absorption becomes bigger, which is ascribed to the enhancement of the quantum confinement effect.

## 2. Materials and Methods

### 2.1. The Preparation of CdTe NC/PVA Films

Luminescent colloidal CdTe NCs are usually synthesized by reproducible one-pot [17,18], precursor noninjection [19], microwave irradiation [20], or non-organometallic phosphine-free synthesis [21]. Aqueous CdTe NCs are synthesized via a facile one-pot synthesis method, in which prepared thiol-capped NCs in water remained essentially the same as reported previously [22]. In a typical synthesis process, 0.4565 g (2.35 mmol) of CdCl_2_·2.5H_2_O was added in 500 mL water, and appropriate thioglycollic acid was dissolved under stirring, followed by changing the pH via the dropwise addition of NaOH solution with 1 mol/L concentration. The obtained solution was transparent and placed in a three-necked flask fixed with a division plate and valves, then deaerated with nitrogen bubbling for 30 min. The prepared 0.612 mL NaHTe was added to the solution together with a slow N_2_ gas flow under stirring. The formation and growth of the NCs progressed upon refluxing at 100 °C with a condenser attached under open conditions [23]. Tuning the PH value (reaction time) of the solution, sample numbers were assigned to CdTe1, CdTe2, CdTe3, and CdTe4 when the PH values (reaction times) were 9 (2 h), 10 (4 h), 11 (10 h), and 11 (12 h), respectively. First, 1 g PVA powder and 10 g deionized water were mixed under stirring at 50 °C for 2 h. Second, the concentration of a series of CdTe NCs was assigned to 0.5% through the controllable addition of CdTe NCs, and uniformly dispersed CdTe NCs/PVA solution was formed after ultrasonication (30 min) and magnetic stirring (30 min). CdTe NC/PVA film can be formed after solution titration, on a clean glass substrate, after dehydration in the vacuum oven. The materials and reagents were purchased from Sinopharm Chemical Reagent Co., Ltd. (Shanghai, China).

### 2.2. Optical Experimental Setup

#### 2.2.1. Temperature-Dependent Steady-State and Time-Resolved PL Measurement

The UV–visible absorption and luminescence spectra of the synthesized CdTe NCs were analyzed on a UV–Vis spectrometer (TU-1901, Persee, Beijing, China) and spectrofluorometer (F-4500, Hitachi, Tokyo, Japan). A series of CdTe NC/PVA films on BK7 glass substrate were fabricated for the temperature-dependent PL measurement. A continuous-wave (CW) laser (MLL-200 mW, CNI, Changchun, China) was used as an excitation light source at 405 nm for steady-state PL measurement. The steady-state PL spectrum was collected by a charge coupled device (CCD) (Synapse, Jobin Yvon, Paris, France) with a spectrometer (IHR550, Horiba, Paris, France). The temperature-dependent PL measurement was performed using a vacuum cryostat (Cryo-77, Oriental Koji, Tianjin, China) with the ability to realize temperature variation from 78 to 300 K. Time-resolved excitonic PL spectra were measured with an intensified CCD (PI-MAX-1024i, Princeton Instruments, NJ, USA) with a spectrometer (SP-2500, Princeton Instruments, NJ, USA), of which time resolution is ~2 ns. Through second-harmonic generation, a femtosecond regenerative amplifier (Legend, Coherent, CA, USA) produces 800 nm laser pulses with 1 kHz repetitive frequency, which are used to change the pulse wavelength at 400 nm as excited pulses.

#### 2.2.2. Femtosecond Z-scan Measurements

Z-scan setup under femtosecond laser excitation has been described previously [24]. The open-aperture Z-scan setup was carried out with femtosecond laser pulses (Legend, Coherent) operating at 800 nm, 1 kHz to avoid heat accumulation. The focused beam waist was estimated to be ~90 μm, and the Rayleigh range *z_0_* was about 1.6 mm, which satisfies the thin-medium condition [25]. The incident and transmitted laser powers were monitored as the samples moved along the laser propagation direction. The open-aperture Z-scan signal was collected by a Silicon detector (Det36A, Thorlabs, NJ, USA) and then processed with a lock-in amplifier (Model 5210, EG&G, TN, USA). The pulse energy was measured via an energy meter (J-10SIHE, Coherent, CA, USA).

## 3. Results and Discussions

### 3.1. Basic Structural and Optical Properites

**Figure 1:**
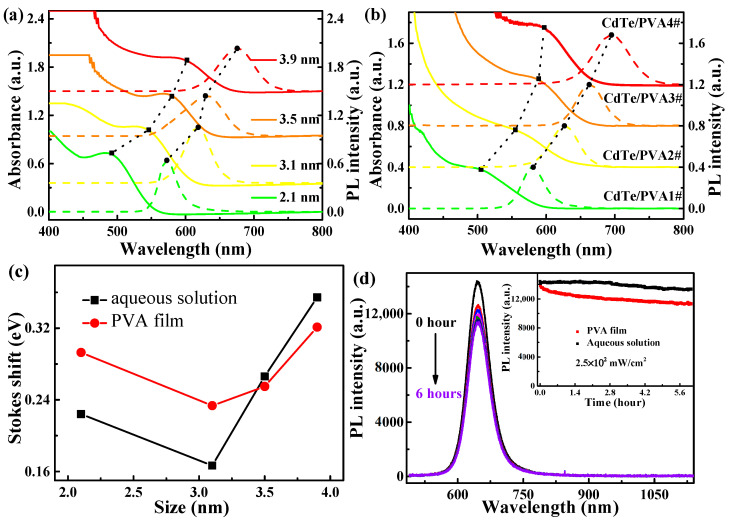
(**a**) UV–visible absorption and PL spectra of aqueous CdTe NCs. (**b**) UV–visible absorption and PL spectra of CdTe NC/PVA films where the solid line is the absorption spectrum, the dashed line is the PL spectrum, and the dot line is the size of the variant absorption and PL peaks. (**c**) Stokes shift of CdTe NCs in aqueous solution and PVA film with the sizes increasing. (**d**) PL spectra of CdTe NC/PVA film with the same experimental conditions. The inset is PL peak intensity of CdTe NCs in aqueous solution and PVA film with the change of radiation time.

Figure 1a,b compares the optical absorption and luminescence spectra of the aqueous CdTe NCs solution and its PVA films at room temperature. The absorption spectra of PVA films indicated a small redshift when compared with aqueous CdTe NCs, demonstrating the ripening of nanoparticles during the polymerization of NCs [26]. The peaks of absorption blue-shifted from the bandgap of bulk CdTe at 1.40 eV. The aqueous solutions and PVA film of CdTe NCs had their corresponding first absorption peaks, respectively, which can be determined by the differential of the absorption spectrum. Similarly, the sizes of NCs were estimated around 2.1, 3.1, 3.5, and 3.9 nm according to the experimental determination of the extinction coefficient [27]. Stokes shift is the difference between the position of the absorption peak and PL maximum. With the sizes of NCs increasing, no obvious size-dependent Stokes shift was observed for CdTe NCs in aqueous solutions and PVA film, as shown in Figure 1c. After film formation, the changed value of the Stokes shift may be ascribed to the participation of interface states in the process of light absorption [28]. 

The PL intensity of CdTe NC/PVA 2# film under CW laser excitation, as a function of radiation time, is shown in Figure 1d. The PL of aqueous NCs solutions was stable photochemically and thermally under laser radiation of 2.5 × 10^2^ mW/cm^2^; the intensity decreased to 92% after 6 h laser radiation. However, PL intensity of CdTe NC/PVA film dramatically reduced for the beginning 1 h and then reduced slowly under the same experimental conditions. The PL peak position had no shift, which suggests the thermal accumulation can be ignored below the laser fluence of 2.5 × 10^2^ mW/cm^2^. With the laser fluence increasing, the thermal effect can lead to the increase in temperature in the composite; thus, the redshift of the emission peak happened at higher temperature [29].

### 3.2. Steady-State PL Spectrum of CdTe NC/PVA Film at the Temperature from 80 to 300 K

**Figure 2:**
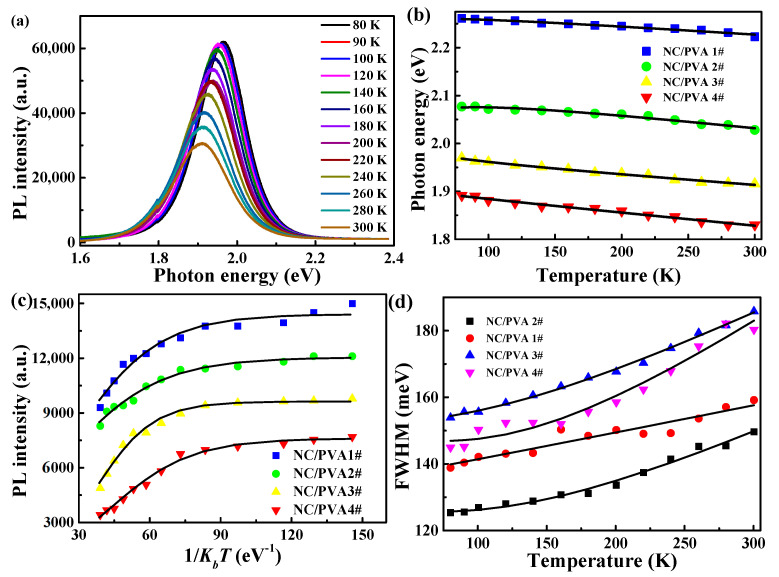
(**a**) The temperature-dependent PL spectra for CdTe NC/PVA 3# film. (**b**) Photon energy of the PL emission peak. (**c**) Integrating PL intensity and (**d**) FWHM for CdTe NC/PVA film; black solid lines are the best-fitted curves based on Equations (1)–(3).

To further study the excitonic recombination of a series of CdTe NC/PVA films, the measurement of steady-state PL was performed. The temperature-dependent steady-state PL spectra of CdTe/PVA 3# are measured and shown in Figure 2a. The temperature-dependent PL peak position, full-width half-maximum (FWHM), and intensity of the NC/PVA films are analyzed. Figure 2b shows the peak position of the PL spectrum, and a clear blue peak shift for excitonic PL can be measured at the temperature change from 80 to 300 K.

For traditional II-VI group semiconductor NCs, with increasing temperature, the PL peak position shifted to a larger photon energy band [30]. The temperature dependence on the bandgap can be expressed by the empirical Varshni equation [31,32] as

(1) $$E_{g}(T)=E_{g}(0)-\alpha \frac{T^{2}}{\left(T+\beta \right)}$$

where *E_g_*(0) is the bandgap at 0 K, *α* is the temperature-dependent coefficient, and *β* is a fitting parameter that is related to the Debye temperature. Figure 2b shows the fitting results for the temperature-dependent PL peaks for the four NC/PVA films. With the size increasing, the obtained temperature coefficient α increased slightly, as shown in Table 1. The fitted α values were in the range of (2.9–3.4) × 10^−4^ eV/K, which is very close to the value of bulk CdTe (0.3 × 10^−4^ eV/K) [33]. The β values changed from 312 to 146 K for CdTe NC/PVA films with larger sizes, which were similar to that of bulk CdTe (160 K) [34], due to lattice softening from bulk to NCs [35]. The fitting results showed that the tendency of the temperature-dependent bandgap was consist with traditional II-VI NCs. As shown in Figure 2c, the excitonic PL intensity decreased with the rising temperatures in CdTe NC/PVA films, and this can be ascribed to the stronger thermal activation process at higher temperatures [36]. The temperature-dependent PL intensity data for the excitonic recombination can be calculated by

(2) $$I(T)=I_{0}/(1+Ae^{-E_{B}/k_{B}T})$$

where *I*_0_ is the initial luminescent intensity, and exciton binding energy (*E_B_*) corresponds to the minimal energy necessary to promote the carrier to the unbound energy level. The term *k_B_T* represents the thermal perturbation, which has a value of 26.0 meV at room temperature. *E_B_* is calculated from Equation (2) by fitting the experiment data 62.7, 54.4, 70.2, and 62.3 meV for a series of CdTe NC/PVA films, respectively, as shown in Table 1. Besides the temperature-dependent PL intensity and bandgap, thermal quenching can also influence the exciton–phonon coupling. The exciton–phonon coupling of CdTe NC/PVA film can be discussed by assessing the temperature dependence of the PL linewidth. The temperature-dependent linewidths of PL emissions for a series of CdTe NC/PVA films is shown in Figure 2d. The monotonous temperature-dependent band broadening can be generally represented as the combination of three terms: linear temperature-dependent, inhomogeneous broadening, and another term expressing homogeneous broadening due to exciton-optical phonon interaction [37]. The temperature dependence on the PL band linewidth is fitted via the Boson equation [38]:

(3) $$\Gamma (T)=\Gamma_{0}+\sigma T+\Gamma_{op}/(e^{ћ\omega_{op}/k_{B}T}-1)$$

where *Γ*_0_ is the inhomogeneous band broadening, and *σT* relates to the homogeneous broadening contribution, which arises from acoustic phonon scattering through the deformation potential with the carrier-acoustic phonon coupling intensity of *σ*. *Γ_op_* is connected with the optical phonon–exciton contribution to the band linewidth broadening, while *ћω_op_* is longitudinal optical–phonon energy, in which *ћ* is the reduced Planck constant and *ω_op_* is the angular frequency of optical phonon oscillation. When the temperature increases, the optical phonon contribution dominates and the band linewidth increases almost linearly with temperature change [39]. The first term is represented in Equation (3) at relatively low temperature, and an approximate constant band broadening is obtained.

### 3.3. Time-Resolved PL Spectra of CdTe NC/PVA Films at Temperatures from 80 to 320 K

**Figure 3:**
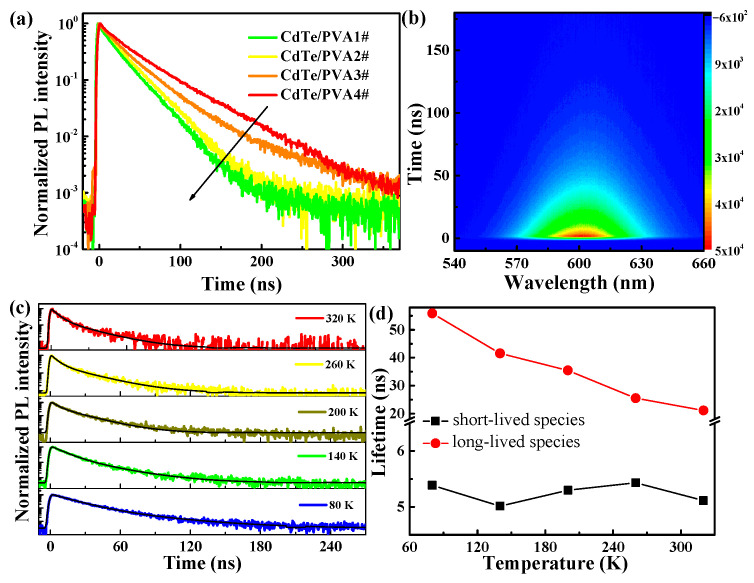
(**a**) PL decays at the emission peak of CdTe NC/PVA films at 290 K. (**b**) Time-resolved PL contour spectra from -10 to 200 ns at 290 K. (**c**) PL decays of CdTe NC/PVA 3# film at different temperatures; the black line is the fitting curve. (**d**) The lifetimes of PL for CdTe NC/PVA 3# film from a double exponential fitting with the temperature change.

To further reveal the mechanism of excitonic recombination, we carried out time-resolved PL measurement, and some results are shown in Figure 3. The exciton PL dynamics of CdTe NC/PVA films are not single-exponential, and the peak shift at different delay times can be observed, demonstrating the existence of several emission centers in the NCs. The PL dynamics at emission peaks were found to be double exponential, based on nonlinear least-squares fitting, which can be calculated [40] as

(4) $$I(t)=A+\int_{0}^{t}\left(Be^{-(t-t\prime )/\tau_{1}}+Ce^{-(t-t\prime )/\tau_{2}}\right)e^{-{\left(t\prime /\tau_{\mathrm{FWHM}}\right)}^{2}}dt^{\prime }$$

where *τ*_1_ and *τ*_2_ are lifetimes of the long- and short-lived excitonic emission species, respectively. A expresses the background of the signal. B and C are the relative intensities of the two species, respectively. The width of the instrumental response function (IRF) *τ*_FWHM_ is recorded at the emission wavelength of the excitation laser pulse. IRF is considered with Gaussian function to convolute with the PL dynamics. The average PL lifetime of the NCs can be calculated as

(5) $$\tau =(B\tau_{1}{}^{2}+C\tau_{2}{}^{2})/(B\tau_{1}+C\tau_{2})$$

With increasing temperature, the lifetimes of the long- and short-lived species for CdTe NC/PVA films are calculated by double-exponential fitting, as shown in Figure 3d. It has been revealed that the long- and short-lived species can be ascribed to the band-edge excitonic and surface-trapped state, respectively [41]. Moreover, it can be observed from Figure 3d that the lifetime of the carrier-trapped state (*τ*_1_) unchanged with the increasing temperature, while the lifetime of excitonic state (*τ*_2_) became longer, from 0.5 ns to 2.3 ns, when the temperature increased from 80 to 320 K. With increasing temperatures, thermal motion becomes more active and thus prevents the recombination of excitons, resulting in an increased PL lifetime [25]. Here, the average PL lifetimes became shorter with increasing temperature for CdTe NC/PVA films. An increase in excitonic PL lifetimes with temperature elevation has been observed in other measurements of CdTe NCs. The double exponential fitting strongly implies the participation of other excited states in this transition process. A defect or surface-related state is commonly observed in II-VI group semiconductor NCs. These energy levels are more likely to be of surface nature. In general, surface-state energy levels have smaller photon-energy emissions due to lower transition probability, relative to excitonic combination [42]. We adopted a long-lived species to investigate the temperature dependence of CdTe NCs because faster species in biexponential decay usually reflect the excitonic recombination process of an electron and a hole. The lifetime (several nanoseconds) of a PL species does not depend on temperature, as shown in Figure 3d. However, the long-lived species dominated at the temperature change from 80 to 320 K, which is attributed to intrinsic excitonic recombination. The lifetime of the long-lived species elongated from 21.1 ns to 55.8 ns with the temperature change from 80 to 320 K. Besides the quantum confinement effect, experimental results indicate that the PL decay of the CdTe NC/PVA film is related to temperature variation.

### 3.4. Nonlinear Absorption by Femtosecond Z-scan

**Figure 4:**
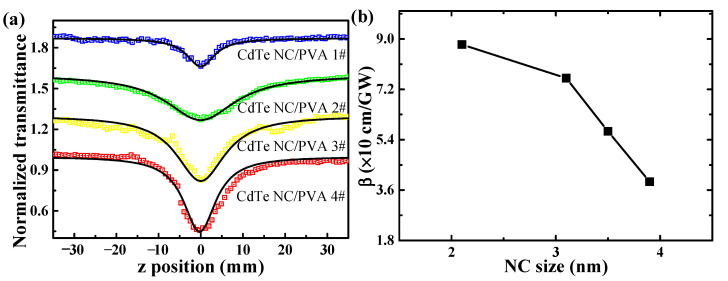
(**a**) Open-aperture Z-scan curves of a series of CdTe NC/PVA films; the black solid lines are the corresponding fitting curves. (**b**) TPA coefficient as a function of NC size in CdTe NC/PVA films.

The nonlinear absorption of NCs in the off-resonant spectral region is important for the potential application in optoelectronic devices [43]. In order to understand the fluence-dependence of nonlinear absorption in CdTe NC/PVA films and discuss the size-dependent influence, open-aperture Z-scan measurements using a femtosecond laser system are performed to reveal the nonlinear absorption mechanisms [44,45]. The experimental results indicate that the NCs expressed reverse saturable absorption effects under femtosecond laser excitation at 800 nm, shown in Figure 4a, as predicted by the theories in reference [44]. The photon energy of one photon at 800 nm is not enough to excite the NCs. Thus, it is reasonable to attribute the nonlinear absorption of CdTe NCs at 800 nm to two-photon absorption (TPA). Upconversion of PL can be observed due to TPA [42], when the film passed the focal point of lens during the motor stage. The nonlinear absorption coefficients were 8.8, 7.6, 5.7, and 3.9 × 10 cm/GW, respectively, which demonstrates that nonlinear absorption coefficients decreased with the increase in NC size. Because the PVA film thickness can be fabricated to hundreds of microns thin, these values of CdTe NC/PVA films are larger by three orders of magnitude than the known values of aqueous CdTe NCs in water (~4 nm–2.5 × 10*^−^*^3^ cm/GW) [42] and CdSeTe NCs in toluene (3.5 nm–3.1 × 10*^−^*^2^ cm/GW) [24]. No signal can be observed for the pure PVA film under the same experimental conditions. It is worth pointing out that the β values of CdTe NC/PVA films are much larger than that of CdTe NCs in solution. According to the results reported, the nonlinear absorption behavior of CdTe NC/PVA films results from free-carrier absorption due to TPA.

## 4. Conclusions

In conclusion, the temperature-dependent spectral properties of CdTe NC/PVA films are studied via PL spectroscopy and Z-scan techniques. From the temperature-dependent PL spectra, a smaller photon energy of the PL emission peak, a rapidly decreasing PL intensity, and a wider linewidth are observed with temperature increasing from 80 to 300 K. It is revealed that the excitonic PL consists of band-edge state and trapped state emissions and, therefore, presents double exponential dynamics. The short-lived emission species is attributed to surface-trapped state recombination in NCs that has a photogenerated trapped channel and a time-resolved peak shift. The species with a long-lived lifetime is ascribed to the excitonic band-edge recombination. Through the femtosecond Z-scan method, the calculated nonlinear two-photon absorption coefficient becomes smaller as the size of the NCs increases. The temperature dependence of the PL for CdTe NC/PVA films reveals the luminescent and thermal behavior, which help us to understand CdTe NC/PVA films, for potential use in display and LED applications.

## Figures and Tables

**Table 1:** Fitted parameters in terms of Equations (1)–(3).

Films	*E_g_*(0) (eV)	*α* (eV/K)	*β* (K)	*E_B_* (meV)	*Γ_0_* (meV)	*Γ_op_* (meV)	*ћω_op_* (meV)
CdTe NC/PVA 1#	2.26	2.9 × 10^−^^4^	312	62.7	16	16	20
CdTe NC/PVA 2#	2.08	3.2 × 10^−4^	244	54.4	25	18	20
CdTe NC/PVA 3#	1.97	3.4 × 10^−4^	233	70.2	42	20	21
CdTe NC/PVA 4#	1.89	3.4 × 10^−4^	146	62.3	54	21	19

## Data Availability

Not applicable.
